# Neuroprotective Effects of a Benzofuran-Containing Selenium in a Mouse Alzheimer’s Disease Model: Molecular, Biochemical, and Behavioral Analyses

**DOI:** 10.1021/acschemneuro.5c00139

**Published:** 2025-06-16

**Authors:** Tácia Katiane Hall, Mariana Parron Paim, Pâmella da Costa, Amanda Rebelo de Azevedo, Vanessa Nascimento, José Sebastião Santos Neto, Fernanda Severo Sabedra Sousa, Tiago Veiras Collares, Fabiana Kommling Seixas, César Augusto Brüning, Cristiani Folharini Bortolatto

**Affiliations:** † Laboratory of Biochemistry and Molecular Neuropharmacology (LABIONEM), Graduate Program in Biochemistry and Bioprospecting (PPGBBio), Chemical, Pharmaceutical, and Food Sciences Center (CCQFA), Federal University of Pelotas (UFPel), Pelotas, Rio Grande do Sul 96010-900, Brazil; ‡ SupraSelen Laboratory, Department of Organic Chemistry, Institute of Chemistry, Federal Fluminense University, Valonguinho Campus, Niterói, Rio de Janeiro 24020-141 Brazil; § LabRMN, Chemistry Institute, Federal University of Goiás, Goiânia, Goiás 74690-900, Brazil; ∥ Laboratory of Cancer Biotechnology, Biotechnology Graduate Program, Technology Development Center, 37902 Federal University of Pelotas, Pelotas, Rio Grande do Sul 96010-610, Brazil

**Keywords:** selenium, memory, streptozotocin, oxidative damage, neuroinflammation

## Abstract

Alzheimer’s disease (AD) is a neurodegenerative disorder mainly characterized by progressive cognitive decline, for which effective treatments remain limited, and selenium is known for its neuroprotective actions. Thus, this study evaluated the neuroprotective effects of the compound 2-(((3-trifluoromethyl)­phenyl­(selenyl)­methyl)-2,3-dihydrobenzofuran (TFSeB) in a streptozotocin (STZ)-induced AD model in male Swiss mice. The animals received intracerebroventricular injections of STZ (3 mg/kg, a neurotoxic agent) to induce cognitive deficits, followed by treatment with TFSeB (1 and 5 mg/kg, intragastrically). Behavioral tests revealed that, like positive control (memantine), the compound TFSeB improved memory performance in the Y-maze, novel object recognition, and passive avoidance tests, suggesting its ability to counteract STZ-induced memory impairments. Biochemical analyses showed that the compound reduced oxidative stress markers in the prefrontal cortex and cerebellum of mice exposed to STZ, including TBARS, ROS, and nitrite levels while increasing NPSH. STZ induced an increase in monoamine oxidase B (MAO-B) activity in the hippocampus and cortex, as well as in acetylcholinesterase (AChE) activity in the cortex and cerebellum, which were reverted by TFSeB. Hippocampal RT-qPCR molecular analyses revealed that TFSeB modulated apoptosis-related proteins by increasing BCL-2 and decreasing BAX expression, favoring neuronal survival. Moreover, TFSeB increased brain-derived neurotrophic factor (BDNF) and nuclear factor erythroid 2 (NRF2), targets associated with neuroprotection. The compound also decreased key inflammatory and neurodegenerative markers, including nuclear factor kappa B (NF-κB), interleukin-6 (IL-6), and glycogen synthase kinase 3 beta (GSK3B). In conclusion, the compound TFSeB demonstrates promising protective effects in a STZ-induced AD model by modulating key neurochemical, oxidative, and neuroinflammatory pathways.

## Graphical abstract

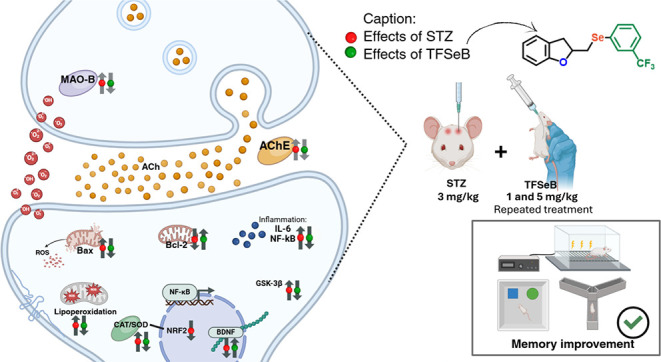

## 1 Introduction

Alzheimer’s disease (AD) represents one of the greatest global public health challenges, affecting millions of people worldwide.^1^ The social impact of the disease is significant, as it progressively compromises individuals’ autonomy, leading to severe cognitive decline, memory loss, difficulties in communication, and impairments in daily activities.^2^ Thus, the search for new therapeutic approaches to slow disease progression and improve patients’ quality of life has become a priority in biomedical research.

AD is a progressive and irreversible neurodegenerative disorder that results from complex processes, including the accumulation of β-amyloid plaques, tau protein hyperphosphorylation, and neuroinflammation, which ultimately lead to synaptic dysfunction and neuronal death.^3^ Moreover, oxidative stress plays a central role in AD pathophysiology, contributing to cellular damage and exacerbating neurodegeneration.^4^ Evidence suggests that increased production of reactive oxygen species (ROS) and reduced cerebral antioxidant capacity contribute to disease progression, promoting inflammatory and apoptotic processes that result in brain atrophy and cognitive dysfunction.^5^

Among the available experimental models for studying AD, memory impairment induced by intracerebroventricular (ICV) injection of streptozotocin (STZ) has been widely used in rodents, as it mimics key aspects of the disease’s pathophysiology, including cognitive deficits, exacerbated oxidative stress, neuroinflammation, and impaired cerebral energy metabolism.^6,7^ STZ is a neurotoxin that disrupts insulin signaling in the brain, leading to metabolic deficits that favor neuronal degeneration.^8^ This model enables the investigation of therapeutic strategies targeting oxidative stress and neuroinflammation, which are critical factors in AD progression.^6^ Additionally, the reduction in cholinergic neurotransmitter levels and impairment of synaptic plasticity observed in this model further support its relevance for studying the disease and developing new therapeutic approaches.^9^

In recent years, compounds with antioxidant, anti-inflammatory, and neurotransmission-modulating properties have been explored as potential therapeutic strategies for AD.^10^ Among these, selenium-containing compounds stand out due to their neuroprotective properties associated with their ability to reduce oxidative stress and regulate inflammatory pathways.^11^ Selenium is an essential element involved in the activity of antioxidant enzymes, playing a fundamental role in protecting against oxidative damage and regulating cellular signaling.^12,13^ In this sense, selenium has a crucial role in maintaining healthy brain activity. Studies have shown that organoselenium compounds can modulate neuroinflammation, attenuate β-amyloid accumulation, and preserve mitochondrial function, which are crucial characteristics for neuroprotection in AD.^14^

The compound 2-(((3-trifluoromethyl)­phenyl­(selenyl)­methyl)-2,3-dihydrobenzofuran (TFSeB) features a benzofuran core, known for its antioxidant properties, and an organoselenium group, recognized for its role in modulating oxidative stress and neuroinflammation.^12,15^ Although this is the first *in vivo* study of TFSeB in the context of AD, the compound was developed through the combination of compounds and structures known for their therapeutic relevance.^16^ Organoselenium derivatives have demonstrated the ability to attenuate oxidative stress, regulate neuroinflammatory responses, and preserve mitochondrial function.^17^ Similarly, dihydrobenzofuran structures are reported to possess antioxidant and antiapoptotic activities,^18^ supporting the rationale for exploring this hybrid molecule (TFSeB) in neurodegenerative models. Some organoselenium compounds may also modulate the activity of enzymes relevant to neurotransmission, such as acetylcholinesterase (AChE) and monoamine oxidases (MAO).^15^ Indeed, selanyl-2,3-dihydrobenzofurans effectively inhibited cerebral MAO-B activity *in vitro*, with compound TFSeB demonstrating the most pronounced effect.^15^ Considering that MAO-B inhibitors act as therapeutic agents for AD, reducing oxidative damage and improving cognitive function, compound TFSeB emerges as a promising candidate for studies focused on developing more effective therapies for AD.

Given this context, the present study aimed to evaluate the therapeutic of compound TFSeB in a mouse AD model induced by STZ, seeking to understand its mechanisms of action. The investigation mainly focused on the modulation of oxidative stress, inflammation, and neurotransmission key aspects.

## 2 Results and Discussion

### 2.1 Compound TFSeB Enhances Spatial Memory in Mice Exposed to STZ in the Y-Maze Test without Affecting Locomotion

The Y-maze is a behavioral test used in rodents to assess cognitive functions, especially working memory and spatial exploration.^19^ As shown in Figure 1, the treatments triggered significant changes in the cognitive parameter assessed in the Y-maze test [*F*_(5, 39)_ = 4.247, *p* = 0.0035] (Figure 1A). STZ group exhibited a significant reduction of the percentage of correct alternations compared with the naive group, indicating deficits in spatial working memory. Importantly, the administration of compound TFSeB at both doses, as well as the positive control memantine (MEM), effectively reversed this impairment, restoring mouse memory performance to control levels. These findings suggest that compound TFSeB exerts a protective effect against STZ-induced cognitive deficits, particularly in spatial working memory, a domain highly associated with hippocampal function, which has been previously seen in other studies with selenic compounds.^20,21^ No significant differences were observed in the total number of entries [*F*_(5, 39)_ = 0.4377, *p* = 0.8194] (Figure 1B).

**Figure 1:**
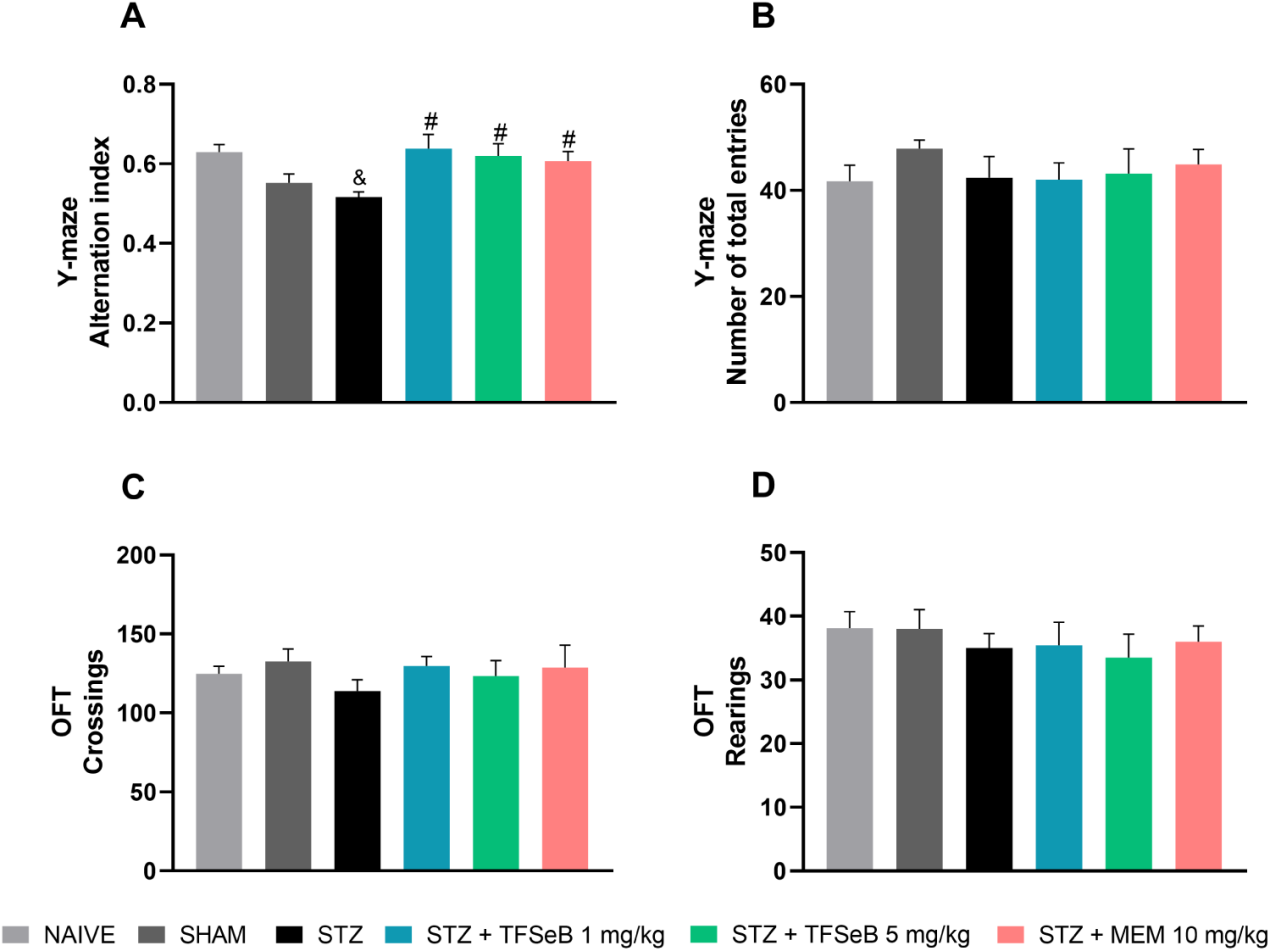
Effects of the compound TFSeB on (A) correct alternation rate and (B) total number of arm entries in the Y-maze test, and (C) crossings and (D) rearings in the open field test (OFT). Values are expressed as the mean ± standard error of the mean (S.E.M.), *N* = 7–8 animals per group. One-way ANOVA followed by Newman–Keuls post hoc test. (&) *p* < 0.05 compared with the naive group. (#) *p* < 0.05 compared with STZ group. Abbreviations: STZ—streptozotocin; MEM: memantine (positive control).

Memory tests may be influenced by the animals’ physical health (sensory or motor functions) and motivation. Due to this reason, an open-field test (OFT) is commonly applied before memory tests, to avoid misinterpretation of results and validate memory tests findings. Our results show that locomotor function in the OFT remained intact in animals exposed to STZ, MEM and/or TFSeB compound with no significant differences in the number of crossings [*F*_(5, 39)_ = 0.2015, *p* = 0.7506] (Figure 1C) or rearings *F*_(5, 39)_ = 0.3570, *p* = 0.8746] (Figure 1D). These findings corroborate the total entries observed in the Y-maze also used as a locomotion parameter.

### 2.2 Compound TFSeB Restores Memory Deficits Caused by STZ in the Object Recognition and Location Tests

Other widely used memory tasks in preclinical research include the new object recognition (ORT) and location (OLT) tests, which assess recognition memory and spatial memory, respectively.^22^ The ORT and OLT followed a similar pattern and are presented in Figure 2. In the training phase, no significant difference was observed in object exploration (data not shown). There were no significant differences between the experimental groups in the total object exploration time in any of the ST-ORT [*F*_(5, 39)_ = 0.7066, *p* = 0.6220] (Figure 2A); LT-ORT [*F*_(5, 39)_ = 0.8117, *p* = 0.5486] (Figure 2D); OLT [*F*_(5, 39)_ = 0.5523, *p* = 0.7356] (Figure 2G), indicating that overall exploratory behavior remained unchanged. However, when assessing exploration time spent on novel object in ST-ORT [*F*_(5, 39)_ = 10.02, *p* < 0.0001] (Figure 2B), LT-ORT [*F*_(5, 39)_ = 12.28, *p* < 0.0001] (Figure 2E) and OLT [*F*_(5, 39)_ = 25.77, *p* < 0.0001] (Figure 2H), differences among groups were detected by one-way analysis of variance (ANOVA). Post hoc demonstrated that STZ impaired animal memory, reducing the time spent exploring the object in all tasks. Notably, administration of compound TFSeB at both doses as well as the positive control (MEM) effectively reversed this impairment, restoring the exploration preference for the novel object or location.

**Figure 2:**
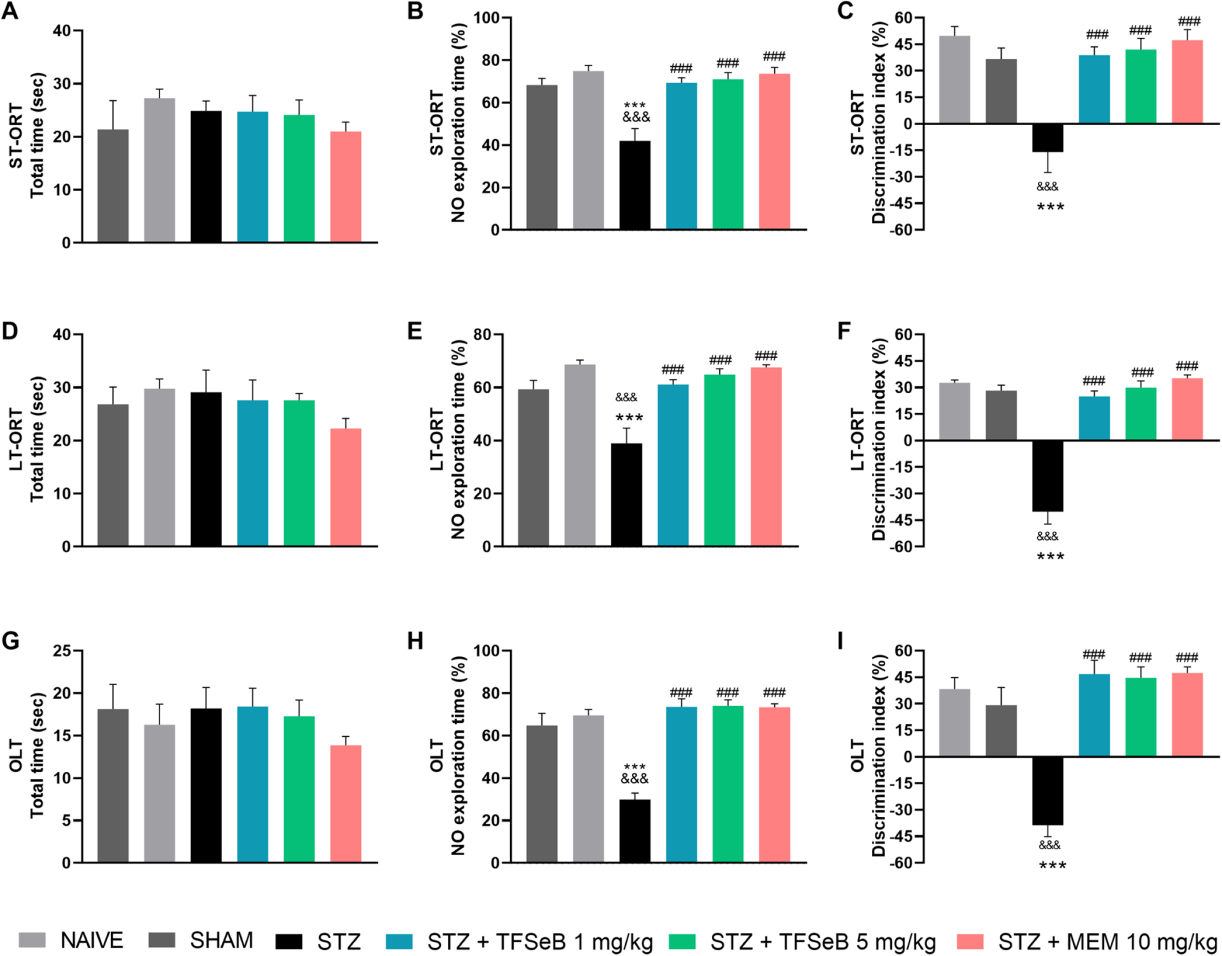
Effects of the compound TFSeB on short-term-object recognition test (ST-ORT), long-term-object recognition test (LT-ORT) and object location test (OLT). Total exploration time of both objects in ST-ORT (A), LT-ORT (D), and OLT (G), respectively. (B, E, H) Exploration time of the novel object in ST-ORT (B), LT-ORT (E) and OLT (H), respectively. Discrimination index in ORT (C), LT-ORT (F) and OLT (I), respectively. Values are expressed as the mean ± standard error of the mean (S.E.M.), 7–8 animals per group. Statistical analysis was performed by one-way ANOVA followed by Newman–Keuls post hoc test. (&&&) *p* < 0.001 compared with naive group; (***) *p* < 0.001 compared with sham group; (###) *p* < 0.001 compared with STZ group. Abbreviations: STZ—streptozotocin; MEM: memantine (positive control).

From the data obtained in these tests it is possible to obtain the discrimination index (DI) that provides a more precise measure of memory performance. A positive DI indicates a preference for the novel object or location, reflecting intact recognition and spatial memory. A negative DI, on the other hand, suggests an inverse preference, meaning the subject explored the familiar object or location more than the novel one, which is indicative of memory impairment.^22^ In the present study, significant alterations were detected by ANOVA for ST-ORT (*F*_(5, 39)_ = 10.20, *p* < 0.0001] (Figure 2C), LT-ORT [*F*_(5, 39)_ = 57.70, *p* < 0.0001] (Figure 2F), and OLT [*F*_(5, 39)_ = 23.66, *p* < 0.0001] (Figure 2I). The STZ group exhibited a significantly negative DI across all tasks, confirming STZ-induced cognitive deficits. Like MEM, treatment with TFSeB at both doses restored the DI to positive values comparable to those of the control groups, indicating a clear reversal of the memory impairment in mice caused by STZ.

### 2.3 Compound TFSeB Improves Memory Performance of STZ Mice in the Stepwise Reduction Inhibitory Avoidance Task

The gradual reduction inhibitory avoidance task, a behavioral experiment used to evaluate learning and fear-associated memory in rodents by assessing their ability to avoid a painful stimulus, was included in our battery of behavioral tests, with results presented in Figure 3. During the training session (Figure 3A), all animal groups exhibited similar behavior by stepping down from the platform, a normal response to the novel environment [*F*_(5, 39)_ = 0.1402, *p* = 0.9818], indicating that learning was consistent across all groups. Upon stepping down, they received a mild electric shock, which served to associate the platform with an aversive stimulus.

**Figure 3:**
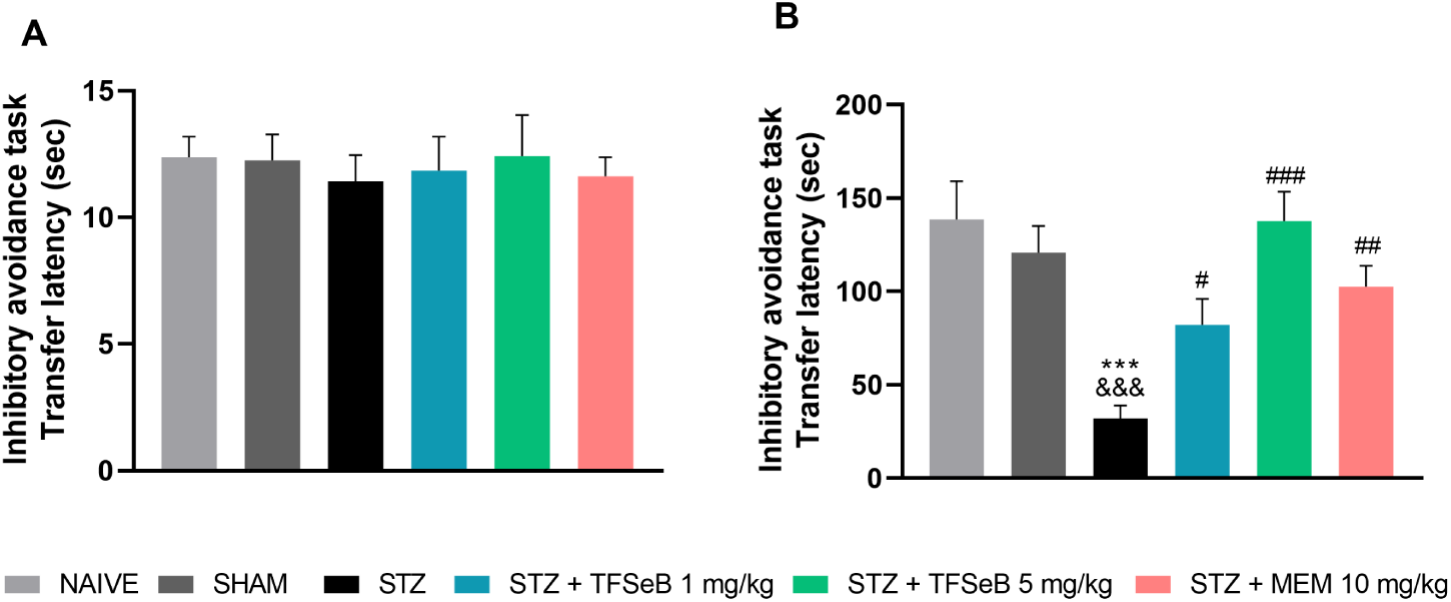
Effects of compound TFSeB on inhibitory avoidance task (A) Latency time to platform descent during training and (B) Latency time on the test day. Data are presented as the mean ± standard error of the mean (SEM), with 7–8 animals per group. Statistical analysis was performed using one-way ANOVA followed by Newman–Keuls post hoc test. (***) *p* < 0.001 compared with naive group; (&&&) *p* < 0.001 compared with sham group; (#) *p* < 0.05, (##) *p* < 0.01, (###) *p* < 0.001 compared with STZ group. Abbreviations: STZ—streptozotocin; MEM: memantine (positive control).

In the test session (Figure 3B), performed 24 h later, animals with intact memory would remember the shock and, as a result, avoid stepping down quickly from the platform. This was reflected in significantly longer step-down latencies observed in the naive and sham groups. Conversely, the group which had impaired memory due to the STZ-induced toxicity displayed a significantly shorter step-down latency, indicating that they failed to remember the previous aversive experience and descended the platform more rapidly. Treatment with compound TFSeB at both doses tested, as well as the positive control (MEM), effectively reversed this memory deficit, with the pharmacologically treated groups showing similar reduction latencies to the naive and sham groups [*F*_(5, 39)_ = 8.226, *p* < 0.0001]. This suggests that TFSeB successfully mitigated the memory impairment induced by STZ, restoring the animals’ ability to recall the previous aversive experience. In addition, the inhibitory avoidance task results were reliable, as all animals exhibited similar behavior during training, and there were no changes in locomotion in the OFT, similar to studies available in the literature with selenium-containing compounds.^23^

### 2.4 Compound TFSeB Normalizes Cortical and Hippocampal MAO-B Activity in STZ Mice

MAO-B plays a crucial role in the activity of monoamines, such as dopamine, and its increased activity is strongly associated with cognitive decline and neurodegenerative diseases. MAO-B inhibitors, such as selegiline and rasagiline, are widely used in the treatment of pathologies such as Alzheimer’s and Parkinson’s due to their ability to preserve dopaminergic neurotransmission and reduce oxidative stress.^24^ Based on previous *in vitro* findings demonstrating that TFSeB inhibits cerebral MAO-B activity,^15^ we extended our investigation to evaluate whether this effect would also occur in *ex vivo* conditions of AD model. To this end, MAO-B activity was analyzed in selected brain regions of treated animals, allowing us to explore the compound’s potential to modulate this enzyme in a physiologically relevant context.

The cortex and hippocampus were explored due to their central role in cognition and memory. The hippocampus is essential for memory consolidation, while the cortex is involved in executive functions and information storage.^25^ Studies indicate severe alterations in these tissues, including dysfunctions in monoaminergic neurotransmission.^26,27^

The results show that STZ administration significantly increased MAO-B activity in the cortex [*F*_(5,39)_ = 4.924, *p* = 0.0014] (Figure 4A) and hippocampus [*F*_(5, 24)_ = 6.42, *p* = 0.0006] (Figure 4D), demonstrating that STZ-induced neurotoxicity impacts the monoaminergic system. Treatment with TFSeB was effective in reversing this change, reducing enzyme activity in the hippocampus (1 and 5 mg/kg) and cortex (5 mg/kg) to levels close to those of the control group. The preservation of monoaminergic activity in these regions may be related to improved synaptic plasticity and memory recovery, given that MAO-B hyperactivity is associated with deficits in long-term potentiation, a fundamental mechanism for memory construction.

**Figure 4:**
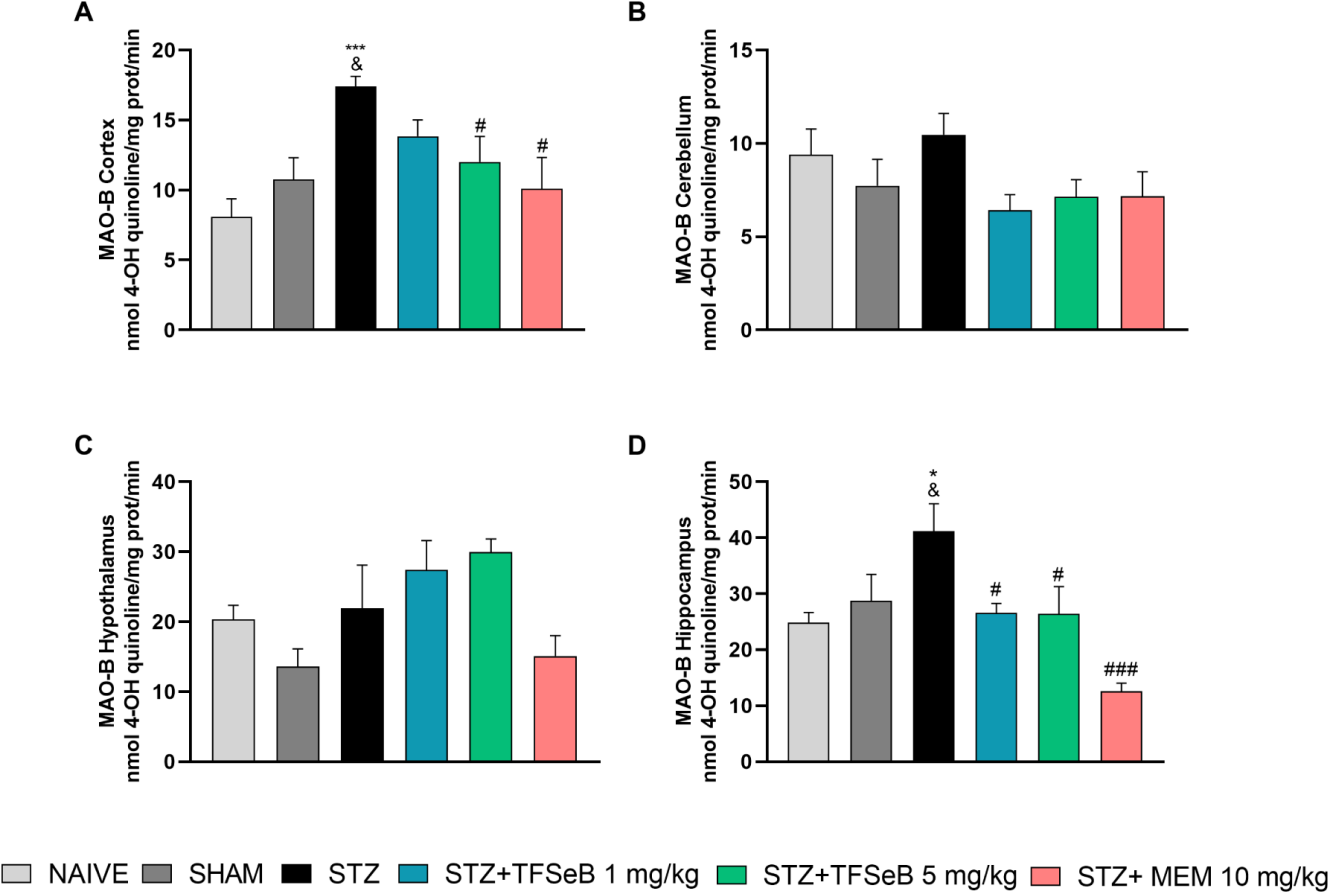
Effects of the compound TFSeB on MAO-B activity in different brain regions: (A) cortex, (B) cerebellum, (C) hypothalamus, and (D) hippocampus. Values are presented as the mean ± standard error of the mean (SEM), with 7–8 animals per group (A, B, and C) or 5 animals per group for (D). Statistical analysis was performed using one-way ANOVA followed by Newman–Keuls post hoc test. (&) *p* < 0.05 compared with the naive group; (*) *p* < 0.05 and (***) *p* < 0.001 compared with the sham group; (#) *p* < 0.05 and (###) *p* < 0.001 compared with the STZ group. Abbreviations: STZ—streptozotocin; MEM: memantine (positive control).

In contrast, no significant differences in MAO-B activity were detected in the cerebellum [*F*_(5, 39)_ = 1.607, *p* = 0.1811] (Figure 4B) and hypothalamus [*F*_(5, 39)_ = 3.000, *p* = 0.0219] (Figure 4C) after STZ administration. Although these regions also play cognitive regulatory roles, the lack of change may indicate that the neurotoxic effect of STZ is more pronounced in the hippocampus and cortex.^17,28,29^

In addition to manipulating neurotransmitters, MAO-B contributes to oxidative stress, as its activity generates hydrogen peroxide (H_2_O_2_), exacerbating neuronal damage.^30^ STZ-induced MAO-B increase may therefore amplify oxidative stress and neuroinflammation, reinforcing neuronal impairment. The reduction of MAO-B activity by TFSeB suggests a dual neuroprotective effect: preservation of monoaminergic neurotransmission and mitigation of reactive oxygen species production. Furthermore, the results corroborate the previously published in vitro MAO-B inhibition results of this compound,^15^ reinforcing the potential of TFSeB as a MAO-B modulator with therapeutic applications in neurodegenerative disorders.

### 2.5 Compound TFSeB Reverses Increased AChE Enzyme in the Cortex and Cerebellum in STZ Mice

AChE is the enzyme responsible for the breakdown of acetylcholine (ACh) in the synaptic cleft, and its hyperactivity is neurotoxic and directly associated with impaired cholinergic neurotransmission—one of the key mechanisms involved in memory loss observed in neurodegenerative diseases.^31^ Statistical analysis of AChE activity revealed a significant impact of the drugs on cortex [*F*_(5, 33)_ = 3.717, *p* = 0.0089] (Figure 5A) and cerebellum [*F*_(5, 39)_ = 11.98, *p* < 0.0001] (Figure 5B). STZ exposure caused increased AChE activity in both structures while treatment with compound TFSeB at both doses effectively normalized its activity. These findings suggest a potential neuroprotective effect of TFSeB, in line with the central role of the cholinergic system in cognition and memory.

**Figure 5:**
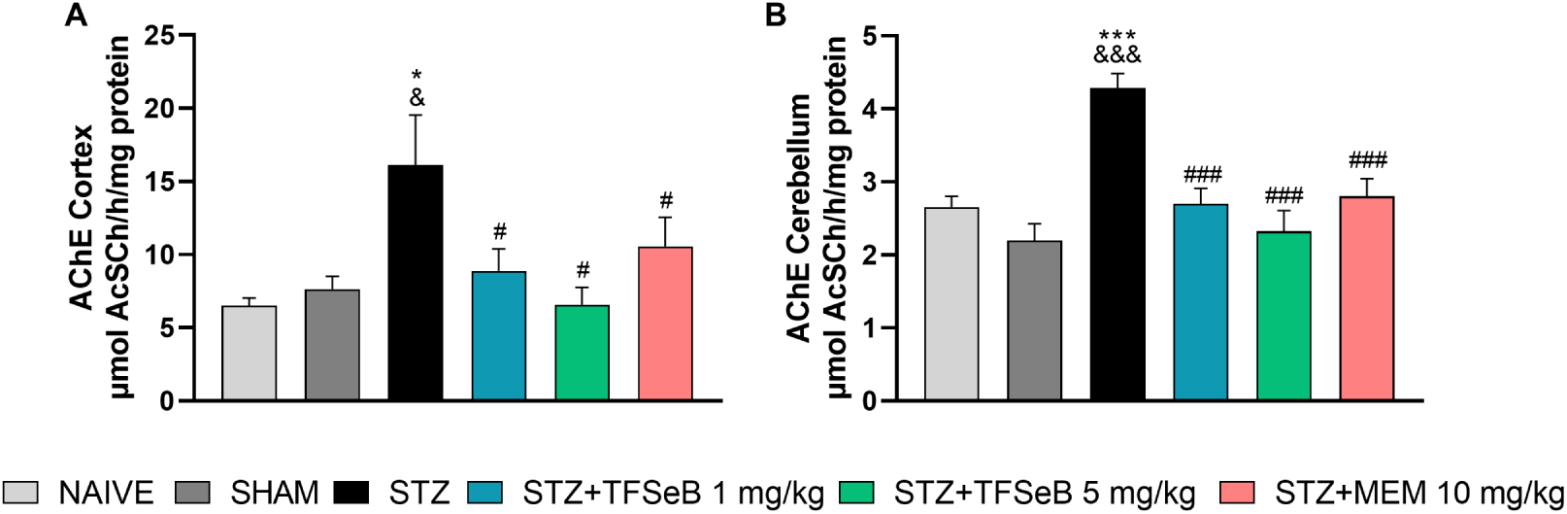
Effects of the compound TFSeB on AChE activity in the (A) cortex and (B) cerebellum. Values are expressed as the mean ± standard error of the mean (SEM), with 7–8 animals per group. Statistical analysis was performed using one-way ANOVA followed by Newman–Keuls post hoc test. (&) *p* < 0.05 and (&&&) *p* < 0.001 compared with the naive group. (*) *p* < 0.05 and (***) *p* < 0.001 compared with the sham group. (#) *p* < 0.05 and (###) *p* < 0.001 compared with the STZ group. Abbreviations: STZ—streptozotocin; MEM: memantine (positive control).

The increase in AChE activity in the STZ group suggests an accelerated degradation of ACh, leading to lower availability of this essential neurotransmitter crucial for synaptic plasticity and learning and memory processes,^32^ which appears to be related to cognitive deficits induced by STZ observed in behavior tests. On the other hand, a reduction in AChE activity observed in the groups treated with the TFSeB compound is a significant finding, as it suggests that this molecule may help restore cholinergic neurotransmission. This is particularly relevant because AChE inhibition is one of the most established therapeutic strategies for AD, forming the basis of drugs such as donepezil and rivastigmine.^33^

Another important aspect is that cholinergic dysfunction does not occur in isolation but rather interacts with other pathological mechanisms, such as oxidative stress and neuroinflammation.^34,35^ In addition to increasing AChE activity, STZ also triggers a neurotoxic environment characterized by the accumulation of ROS and the activation of inflammatory pathways, including nuclear factor kappa B (NF-κB) and pro-inflammatory cytokines.^36^ Therefore, the ability of TFSeB to reduce AChE activity may be associated with broader modulation of these neurodegenerative processes, ultimately contributing to improved synaptic function and cognitive performance, as observed in behavioral tests.

### 2.6 Compound TFSeB Improves Levels of Oxidative Stress Markers in STZ Mice

The brain is highly susceptible to oxidative damage due to its high metabolic demand and lipid-rich composition, making lipid peroxidation a major contributor to neurodegeneration,^37^ and oxidative stress that plays a significant role in the development and progression of AD by contributing to neuronal damage and dysfunction.^4^ For this reason, here, redox parameters such as thiobarbituric acid reactive substances (TBARS), reactive species (RS), NOX and NPSH levels were assessed in the cortex and cerebellum. As represented in Figure 6, statistical analyses revealed significant STZ-induced alterations and protective antioxidant effects of the TFSeB compound.

**Figure 6:**
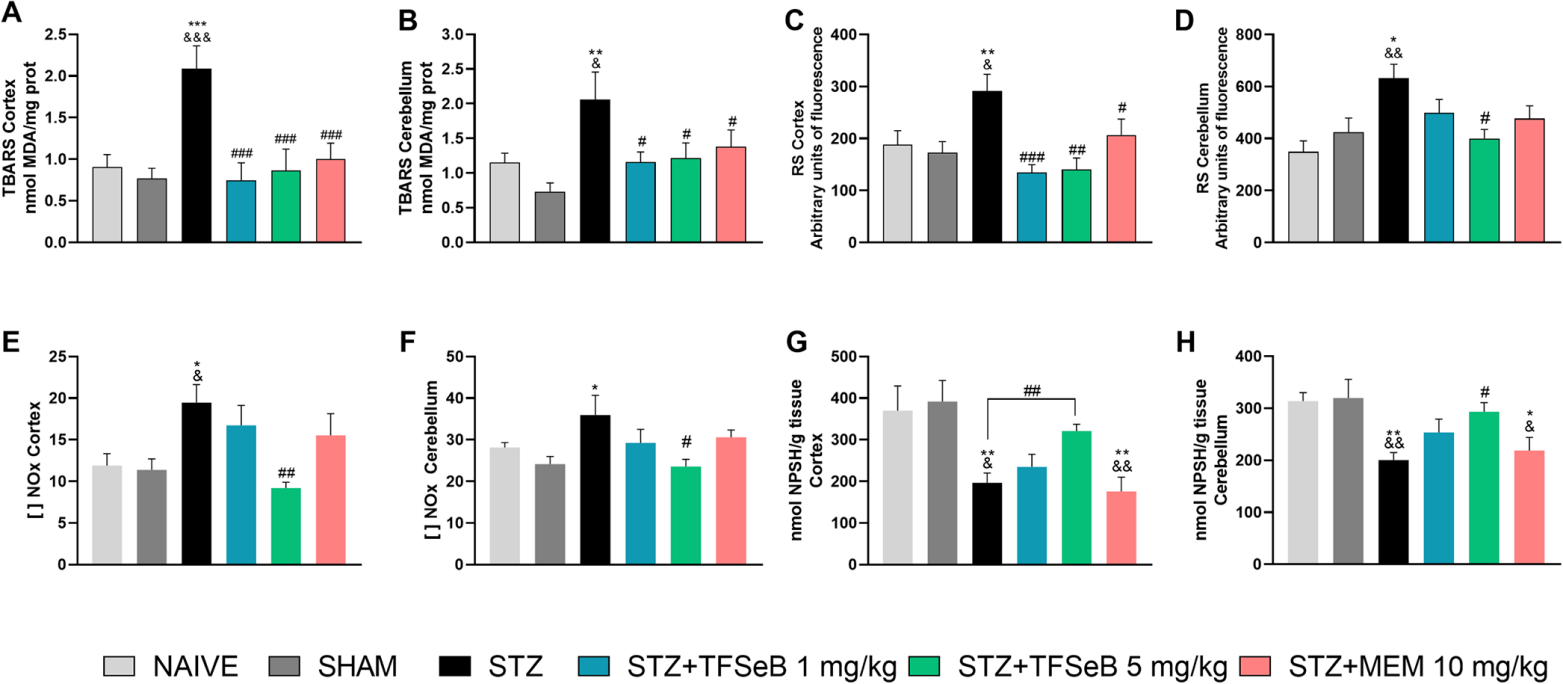
Effects of the compound TFSeB on redox markers (TBARS, ROS, NOx, and NPSH) in the cortex and cerebellum. (A) and (B) represent TBARS levels in the cortex and cerebellum, respectively. (C) and (D) show ROS levels in the cortex and cerebellum. (E) and (F) illustrate NOx levels in the cortex and cerebellum, while (G) and (H) depict NPSH levels in the cortex and cerebellum. Values are expressed as the mean ± standard error of the mean (SEM), with 7–8 animals per group. Statistical analysis was performed using one-way ANOVA followed by the Newman–Keuls post hoc test. (&) *p* < 0.05, (&&) *p* < 0.01, and (&&&) *p* < 0.001 compared with the naive group; (*) *p* < 0.05, (**) *p* < 0.01, and (***) *p* < 0.001 compared with the sham group; (#) *p* < 0.05, (##) *p* < 0.01, and (###) *p* < 0.001 compared with the STZ group. Abbreviations STZ—streptozotocin; MEM—memantine (positive control).

In the lipid peroxidation assay, the STZ group exhibited a pronounced increase of TBARS levels, indicating increased oxidative damage in the cortex [*F*_(5, 39)_ = 6.150, *p* = 0.0003] (Figure 6A) and cerebellum [*F*_(5, 39)_ = 3.727, *p* = 0.0075] (Figure 6B). On the other hand, treatment with TFSeB, at both doses, significantly reduced TBARS levels. This suggests that TFSeB effectively counteracted lipid peroxidation in these brain regions.

Regarding RS production, the STZ group showed a substantial increase in RS levels, potentially related to oxidative damage observed. In the cortex, both doses of TFSeB significantly reduced RS levels [*F*_(5, 39)_ = 5.325, *p* = 0.0008] (Figure 6C). However, in the cerebellum, only the 5 mg/kg dose of TFSeB was able to significantly decrease RS levels [*F*_(5, 33)_ = 4.244, *p* = 0.0043] (Figure 6D). MEM, a *N*-methyl-d-aspartate (NMDA) receptor antagonist, was effective in reducing TBARS and RS in both brain structures, which could be related with its glutamatergic mechanisms.

The observed increase in TBARS and RS levels in the STZ group is consistent with the well-documented role of STZ in inducing oxidative stress and lipid peroxidation in the brain.^38^ STZ is known to generate RS through multiple pathways, including mitochondrial dysfunction, activation of pro-oxidant enzymes, and inhibition of antioxidant defense systems.^6,35^ The resulting oxidative imbalance leads to lipid peroxidation, causing structural and functional damage to neuronal membranes.^39^ The elevated TBARS levels observed in the STZ group provide direct evidence of this oxidative insult, reinforcing the link between STZ-induced neurotoxicity and excessive lipid peroxidation. A key consequence of increased oxidative stress is neuronal dysfunction and apoptosis, particularly in regions critical for cognitive processing, such as the cortex and cerebellum.^40^ The significant reduction in TBARS levels following TFSeB treatment suggests that this compound exerts a protective effect against lipid peroxidation, potentially by enhancing antioxidant defenses or directly scavenging RS. The decrease in RS levels observed in the cortex (both doses) and, to a lesser extent (5 mg/kg), in the cerebellum following TFSeB treatment highlights the region-specific nature of oxidative stress modulation. This suggests that different brain regions may exhibit varying susceptibility to oxidative stress and may require different thresholds of antioxidant intervention for effective protection.^41^ The ability of TFSeB to lower RS levels is particularly relevant, as excessive RS production not only damages cellular structures but also activates inflammatory signaling pathways, further exacerbating neurotoxicity.^42^ By reducing lipid peroxidation and RS accumulation, TFSeB may help preserve membrane integrity, sustain neuronal viability, and maintain synaptic function.

Complementing the oxidative stress analysis, NOx and NPSH levels were also measured in the prefrontal cortex and cerebellum. NO is synthesized by nitric oxide synthases (NOS), with inducible NOS (iNOS) playing a crucial role in neurodegenerative conditions and can be indirectly measured by nitrite and nitrate levels (NOx). Overproduction of NO leads to the formation of peroxynitrite, a highly reactive and neurotoxic species that exacerbates oxidative damage, mitochondrial dysfunction, and neuronal loss.^43^ In parallel, nonprotein sulfhydryl (NPSH) groups refer to free thiol (−SH) groups, mainly from compounds like glutathione (GSH) and cysteine, and are commonly measured as an indicator of antioxidant capacity and redox status.^44^ The statistical analysis of nitric oxide metabolites (NOx) and NPSH in the cortex and cerebellum revealed significant alterations induced by STZ and the potential modulatory effects of TFSeB.

In the cortex [*F*_(5, 39)_ = 3.780, *p* = 0.0069] and cerebellum [*F*_(5, 39)_ = 2.719, *p* = 0.0335], NOx levels were significantly elevated in the STZ group compared with the naive group, while treatment with TFSeB at 5 mg/kg significantly reduced these levels, suggesting a protective effect against STZ-induced disruptions in nitric oxide homeostasis, as can be seen in Figure 6E,F. The increase in NOx levels in the STZ group is consistent with STZ’s ability to induce neuroinflammation and oxidative stress, both of which contribute to excessive nitric oxide (NO) production.^45^ The reduction in NOx levels by TFSeB suggests that this compound may counteract these pathological effects, potentially by inhibiting iNOS expression or enhancing the scavenging of NO-derived radicals, thereby limiting peroxynitrite formation. Given the interplay between NO signaling and neuroinflammation, this effect may also contribute to broader neuroprotective mechanisms.

In addition, STZ administration significantly reduced NPSH levels in both the cortex and cerebellum, highlighting a depletion of endogenous antioxidant defenses. NPSH levels were significantly reduced by STZ in both the cortex and cerebellum compared to the naive and sham groups. However, treatment with TFSeB at 5 mg/kg restored these levels in the cortex (*t* = 4.204, *p* = 0.001, unpaired *t*-test) and cerebellum [*F*_(5, 39)_ = 4.788, *p* = 0.0017], as illustrated in Figure 6G,H. These findings suggest that TFSeB may enhance cellular antioxidant capacity, counteracting the oxidative stress induced by STZ. The depletion of NPSH in the STZ group aligns with its role in redox homeostasis, as nonprotein thiols, primarily represented by glutathione (GSH), are essential for neutralizing reactive oxygen and nitrogen species and maintaining cellular antioxidant defenses.^44,46^ GSH plays a central role in peroxide detoxification and redox balance modulation through the glutathione peroxidase and glutathione reductase systems.^44^ The observed NPSH reduction suggests impaired glutathione metabolism, which may exacerbate oxidative damage and contribute to neurodegeneration. This effect is particularly relevant in the context of neurodegenerative diseases, where GSH depletion is a hallmark of neuronal vulnerability.

### 2.7 Compound TFSeB Regulates Gene Expression Markers in the Hippocampus of STZ Mice

STZ-induced neurodegeneration has been widely associated with memory impairment and pathological processes similar to those observed in AD, emphasizing the importance of understanding the mechanisms by which TFSeB may mitigate these alterations. Figure 7 presents the analyses of gene expression of markers related to oxidative stress, neuroinflammation, neurotrophic support, and apoptosis, conducted on hippocampal samples to provide insights into the molecular effects of STZ and the potential neuroprotective properties of TFSeB (evaluated at 5 mg/kg).

**Figure 7:**
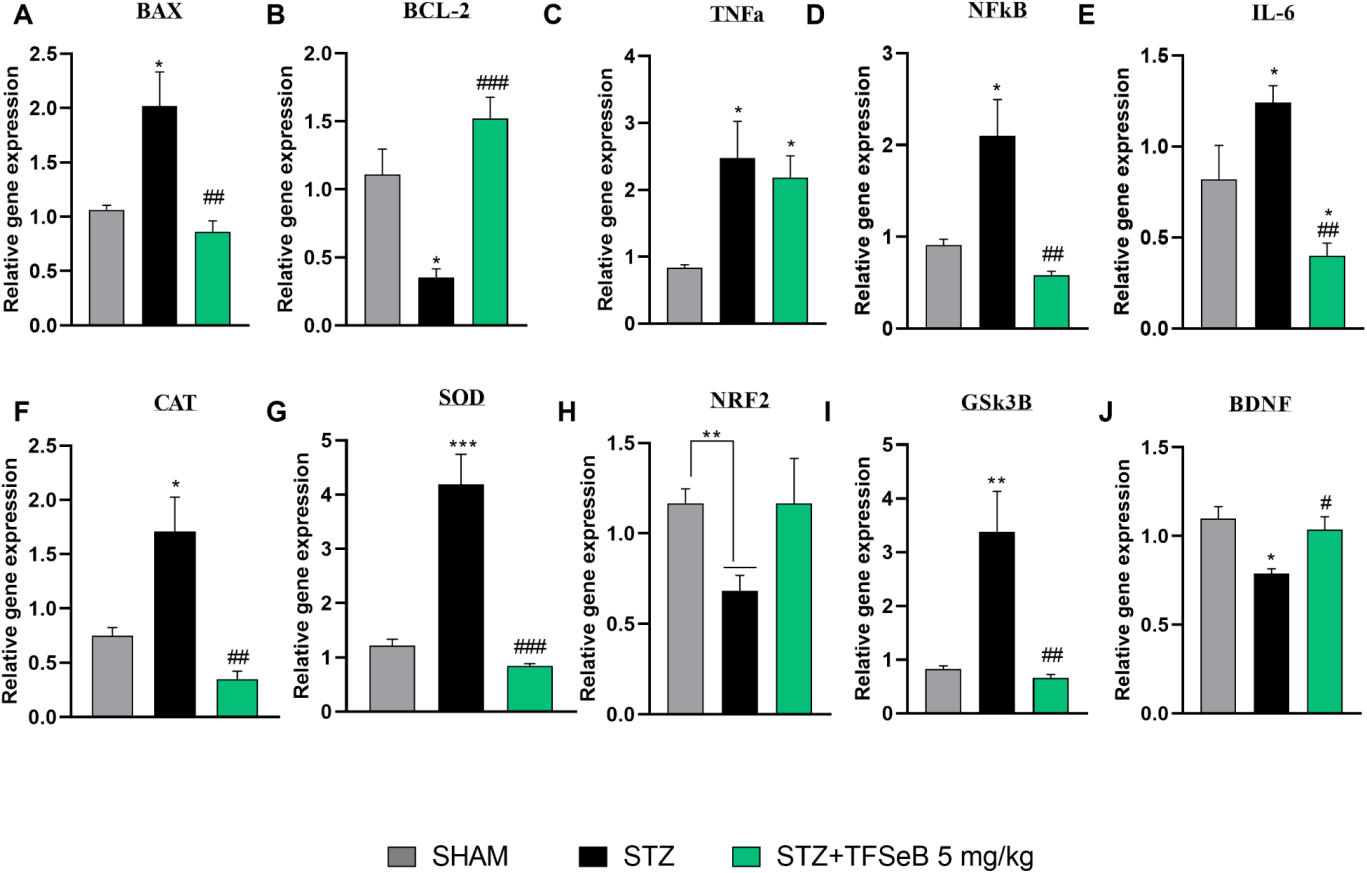
Effects of the compound TFSeB (5 mg/kg) on hippocampal gene expression: mRNA levels of (A) BAX, (B) BCL2, (C) TNF-α, (D) NF-κB, (E) IL-6, (F) CAT, (G) SOD, (H) NRF2, (I) GSK3b, and (J) BDNF. Data are presented as the mean ± standard error of the mean (SEM); *n* = 4 per group. Statistical analysis was conducted using one-way ANOVA followed by the Newman–Keuls post hoc test (*) *p* < 0.05, (**) *p* < 0.01, and (***) *p* < 0.001 represent comparisons with the sham group; (#) *p* < 0.05, (##) *p* < 0.01, and (###) *p* < 0.001 represent comparisons with the STZ group, except for NRF2, which was analyzed using an unpaired *t* test (**) *p* < 0.01.

Oxidative stress plays a critical role in neurodegeneration, and STZ administration led to a decrease in NRF2 expression^47^ [*F*_(2, 9)_ = 3.099, *p* = 0.0946] (Figure 7H), suggesting an impaired antioxidant response. Although treatment with TFSeB increased NRF2 expression, this effect did not reach statistical significance. However, this does not exclude the possibility that a higher dose or prolonged exposure could enhance NRF2 activation to a significant level. In parallel, STZ induced a compensatory increase in the activity of antioxidant enzymes, CAT [*F*_(2, 9)_ = 13.29, *p* = 0.002] (Figure 7F) and SOD [*F*_(2, 9)_ = 30.72, *p* < 0.0001] (Figure 7G), likely as a response to excessive ROS.^45^ Treatment with TFSeB reduced the activity of both enzymes, bringing them closer to sham levels, suggesting a decrease in oxidative burden. These findings align with the hypothesis that TFSeB exerts antioxidant effects, possibly by modulating intracellular redox pathways such as NRF2/ARE or directly neutralizing ROS.^45^

Neuroinflammation is another key factor in AD pathology. STZ administration significantly increased NF-κB expression (Figure 7D), a major transcription factor mediating inflammatory responses^48^ [*F*_(2, 9)_ = 11.83, *p* = 0.0030], as well as IL-6 levels [*F*_(2, 9)_ = 11.13, *p* = 0.0037] (Figure 7E). Treatment with TFSeB effectively attenuated these effects, reinforcing its anti-inflammatory potential. Similarly, TNF-α, a critical cytokine in AD progression,^49,50^ was elevated in the STZ group [*F*_(2, 9)_ = 5.653, *p* = 0.0257], and although TFSeB treatment led to a reduction, this decrease was modest (Figure 7C), suggesting that the compound’s effects on neuroinflammation may be more selective or dependent on additional regulatory mechanisms.^51–53^

The inflammatory response triggered by STZ also impacted on neurotrophic support. BDNF expression, essential for synaptic plasticity and memory consolidation, was significantly reduced in the STZ group (Figure 7J), reflecting the cognitive impairments observed in AD models.^54,55^ Treatment with TFSeB restored BDNF levels [*F*_(2, 9)_ = 16.79, *p* = 0.0009], suggesting a potential role in promoting neuroplasticity. This effect may be linked to intracellular signaling pathways such as CREB and Akt, which regulate neuronal survival and have been explored as therapeutic targets in AD.^55^

Finally, apoptotic markers reflected the downstream consequences of oxidative and inflammatory stress. The pro-apoptotic protein BAX was significantly upregulated in the STZ group compared with the sham group, whereas TFSeB treatment effectively reduced its expression [*F*_(2,9)_ = 10.30, *p* = 0.0047] (Figure 7A). Conversely, BCL-2, an essential antiapoptotic protein, was downregulated in the STZ group but restored by TFSeB treatment [*F*_(2,9)_ = 16.79, *p* = 0.0009] (Figure 7B). These results indicate that TFSeB may help maintain mitochondrial integrity and inhibit apoptosis, further supporting its neuroprotective effects.^56^

Overall, these findings reinforce the hypothesis that TFSeB modulates oxidative stress, neuroinflammation, and apoptosis, key processes involved in AD-related neurodegeneration. The restoration of BDNF levels, along with the regulation of inflammatory and apoptotic markers, suggests that TFSeB contributes to neuronal survival and synaptic plasticity.

### 2.8 Repeated Treatment with Compound TFSeB Does Not Induce Toxicity Effects

Analysis of alanine aminotransferase (ALT) and aspartate aminotransferase (AST) activities, as well as urea levels, indicated that chronic treatment with compound TFSeB did not cause significant changes in markers of liver and kidney function. The results for ALT [*F*_(5, 39)_ = 0.4875, *p* = 0.7835] (Figure 8A) and AST [*F*_(5, 39)_ = 0.6271, *p* = 0.6801] (Figure 8B) showed no differences between the groups, which evidenced the absence of hepatotoxicity. Likewise, urea levels [*F*_(5, 47)_ = 0.6941, *p* = 0.6305] remained unchanged, suggesting that the treatment did not negatively affect the kidneys (Figure 8C).

**Figure 8:**
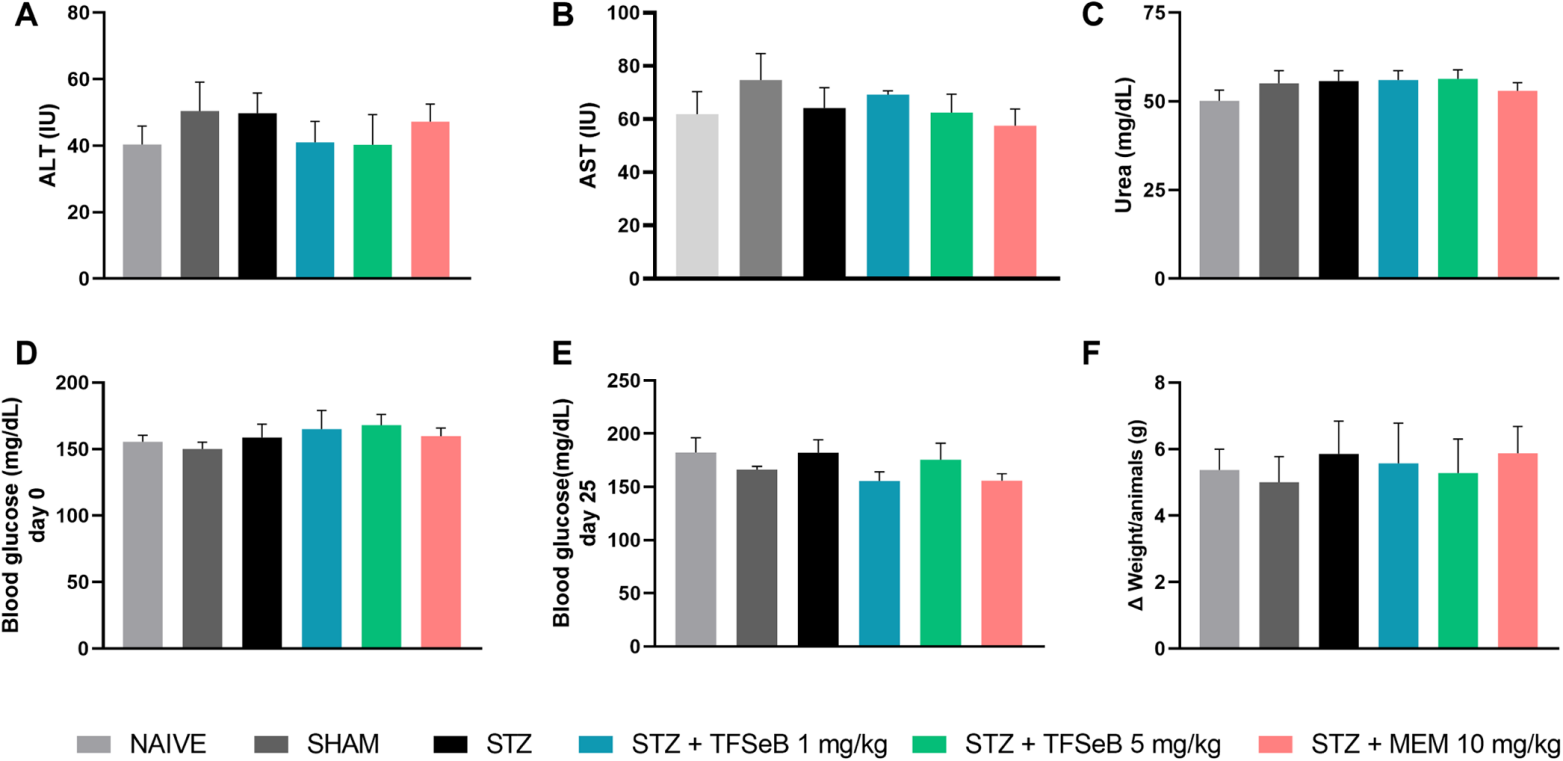
Effects of the compound TFSeB on (A) ALT, (B) AST, (C) Urea, (D) Blood glucose on day 0, (E) Blood glucose on day 25, and (F) Weight Variation. Data are presented as the mean ± standard error of the mean (SEM), with 7–8 animals per group. No significant differences were observed between groups. Statistical analysis was performed using one-way ANOVA followed by the Newman–Keuls post hoc test. Abbreviations: STZ—streptozotocin; MEM: memantine (positive control).

Furthermore, no significant changes were observed in blood glucose levels before [*F*_(5, 39)_ = 1.146, *p* = 0.3530] (Figure 8D) and after treatment [*F*_(5, 39)_ = 1.333, *p* = 0.2707] (Figure 8E) with TFSeB or in the body weight of the animals [*F*_(5, 39)_ = 0.1468, *p* = 0.9798] (Figure 8F), reinforcing the safety of the TFSeB compound in relation to these parameters.

These findings are essential for assessing the safety of the compound TFSeB, especially in a chronic treatment context. Liver and kidney integrity are essential to ensure that the metabolism and elimination of the compound occur without adverse effects, while the stability of blood glucose levels and body weight indicates that the treatment did not interfere with the metabolic balance of the animals. Thus, the results reinforce the safe profile of TFSeB, with no evidence of relevant toxicity from repeated administrations at low doses that could compromise its potential therapeutic use. Additionally, future studies employing standardized protocols for acute oral toxicity at higher doses *in vivo* may provide further insights into the compound’s safety profile and tolerability limits.

In addition to toxicity profile, the characterization of pharmacokinetic parameters represents a fundamental step in the drug development process, especially for compounds intended for oral administration, as these properties critically determine therapeutic efficacy, safety, and optimal dosing strategies. Key ADME properties, such as intestinal absorption, metabolic stability, distribution profile, and blood–brain barrier permeability, must be carefully characterized to ensure that the compound reaches its target site at appropriate concentrations without inducing systemic toxicity. Therefore, we acknowledge that the absence data on the pharmacokinetic properties of the compound TFSeB represents a limitation of the present study. In addition, we recognize the importance of studying the MAO selectivity, extending this investigation to both sexes, well as exploring AD comorbidities (e.g., psychiatric diseases). Together, these future findings may provide important preclinical support for the continued development of compound TFSeB as a neuroprotective candidate.

## 3 Conclusions

Through behavioral, biochemical and molecular approaches, this study demonstrated that the compound TFSeB exerted protective effects in a model of STZ-induced AD. Treatment with TFSeB modulated processes involved in STZ toxicity, including neuroinflammation, oxidative stress, synaptic dysfunction, in addition to restoring the levels of mRNA of proteins essential for neuroplasticity and cognitive function. These effects are in line with previous investigations on selenium compounds, reinforcing their therapeutic potential. Thus, TFSeB shows itself to be a promising candidate for the development of new strategies in the treatment of neurodegenerative diseases. Additional studies evaluating the pharmacokinetic properties, additional oral safety as well as potential efficacy of TFSeB in the treatment of AD comorbidities such as anxiety and depression, will be crucial to advancing its therapeutic development.

## 4 Materials and Methods

### 4.1 Drugs

The compound 2-(((3-trifluoromethyl)­phenyl (selenyl)­methyl)-2,3-dihydrobenzofuran (TFSeB) (Scheme 1 and Figure 9) was previously synthesized and characterized by the SupraSelen Laboratory of the Fluminense Federal University (UFF) using a solvent- and metal-free methodology, through a reaction between 2-allylphenol and diphenyl diselenide, catalyzed by I_2_/DMSO under microwave-assisted heating (Scheme 1).^1^ Due to its lipophilic nature, the compound TFSeB was dissolved in canola oil for intragastric (i.g.) administration.

**Scheme 1:**
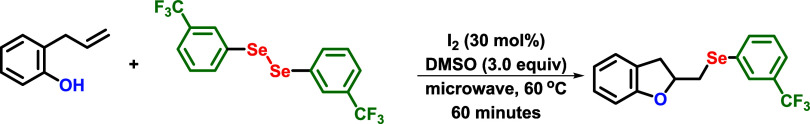
General Synthesis of 2-(((3-Trifluoromethyl)­phenyl­(selenyl)­methyl)-2,3-dihydrobenzofuran (TFSeB)^1^

**Figure 9:**
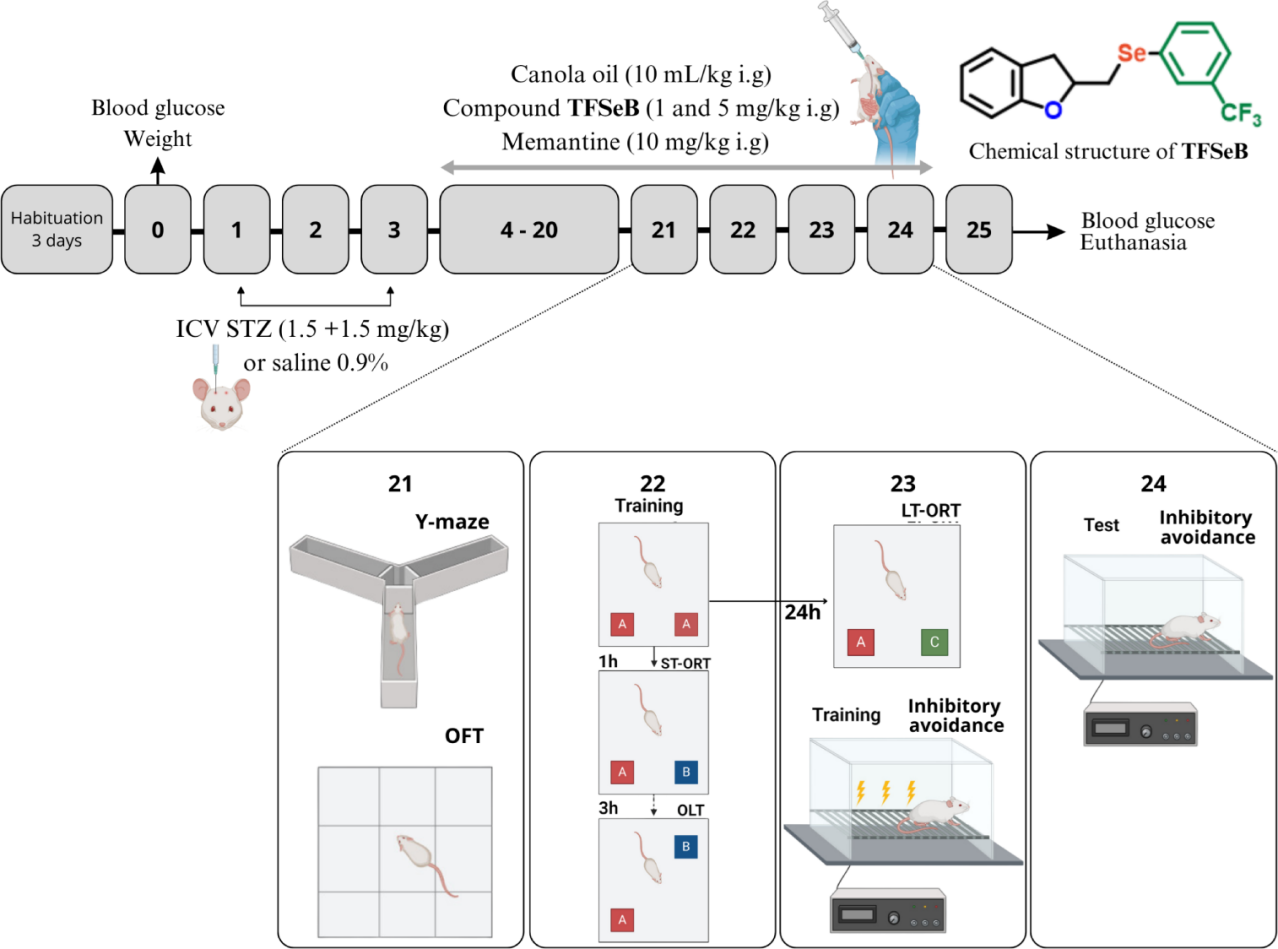
Experimental design and chemical structure of the compound TFSeB. ICV: intracerebroventricular injection; STZ: streptozotocin; OFT: open field test; LT-ORT: long-term object recognition test; ST-ORT: short-term object recognition test; OLT: object location test.

Streptozotocin (STZ), purchased from Sigma-Aldrich, was prepared in a 0.9% saline solution and administered via intracerebroventricular (ICV) injection at a cumulative dose of 3 mg/kg, in a fixed volume of 3 μL per ventricle. Memantine, obtained from commercial suppliers, was dissolved in a 0.9% saline solution and administered via i.g. In all experiments, treatments were administered daily in a constant volume of 10 mL/kg of body weight.

### 4.2 Animals

Male Swiss mice (25–35 g) were obtained from the Central Animal Facility of the Federal University of Pelotas (UFPel) and housed under controlled conditions of temperature (22 ± 1 °C) and a 12 h light/dark cycle, with ad libitum access to water and standard chow. All experimental procedures were approved by the Animal Ethics Committee (CEUA) of UFPel (approval number 032391/2023-84) and conducted in accordance with the guidelines of the National Council for the Control of Animal Experimentation (CONCEA).

### 4.3 ICV Administration Procedure

The solution (STZ or saline) was administered into the lateral ventricles via free-hand ICV injection using a 27-gauge hypodermic needle attached to a Hamilton microsyringe. To ensure accurate depth, the needle was marked and fitted with a rubber stopper. The system was sterilized with 70% alcohol, and a caliper was used for precise measurement. Injections were performed on days 1 and 3 of the experimental protocol, following the anatomical coordinates from the Franklin and Paxinos atlas:^57^ AP = −0.1 mm, ML = 1 mm, DV = 3 mm.

### 4.4 Experimental Protocol (AD Induction Model)

After 3 days of acclimatization, male Swiss mice (*n* = 7–8 per group) were divided into six experimental groups:

- Naive (no procedures or treatments)
- Sham (ICV saline 0.9% + i.g., canola oil)
- STZ (ICV + canola oil)
- STZ + Compound TFSeB (1 mg/kg)
- STZ + Compound TFSeB (5 mg/kg)
- STZ + Memantine (10 mg/kg)

Experimental design is represented in Figure 9. On day 0, body weight and basal blood glucose levels were recorded. On days 1 and 3, the animals were anesthetized and received ICV injections of STZ (total: 3 mg/kg, administered as 1.5 mg/kg per day) or saline solution, in a volume of 3 μL bilaterally.^58^ The ICV-STZ route was selected since it is a well-established model for inducing cognitive deficits, providing direct access to the brain and ensuring the accurate reproduction of symptoms characteristic of sporadic AD, with minimal peripheral interference.

Between days 4 and 24, the animals received daily oral treatments (i.g.) with the compound TFSeB at doses of 1 and 5 mg/kg or memantine (10 mg/kg, a NMDA receptor blocker) which served as a positive control. TFSeB was administered orally (i.g.) to mimic a clinically relevant, noninvasive route, consistent with the oral administration of memantine in clinical settings. Sham and STZ groups received canola oil, the vehicle used for TFSeB, via i.g., administration to ensure uniform vehicle exposure across experimental conditions. The naive group did not receive any treatment, either oral or ICV.

Behavioral tests were performed between days 21 and 24 to assess locomotor and exploratory activity, as well as the learning and memory capabilities of each group. On day 25, the mice were euthanized with isoflurane followed by cervical dislocation, and their brains were dissected for cortex, cerebellum, hippocampus, and hypothalamus collection which were intended for subsequent biochemical analysis (Figure 9).

Due to tissue limitations, the hippocampal structure samples were divided between the MAO-B activity assay and RT-qPCR analyses only, while the hypothalamus was used exclusively for the MAO-B activity assay. The cortex and cerebellum were processed for all biochemical analyses, except for RT-qPCR.

### 4.5 Behavioral Tests

#### 4.5.1 Open Field Test

The mice were subjected to the OFT in a wooden apparatus (30 × 30 × 15 cm) divided into nine equal quadrants, for a period of 5 min. Locomotor activity was quantified by the number of quadrants crossed with all four paws (crossings), while exploration was measured by the frequency of rearing on the hind limbs.^59^ In addition to behavioral assessment, this test also served as a habituation phase for the subsequent novel object recognition test (ORT).

#### 4.5.2 Y-Maze Test

Mice were subjected to the Y-maze test to assess spatial memory and decision-making.^19^ The apparatus consists of three arms arranged at 120° from each other. Each animal was placed in one of the arms of the maze and had 6 min to explore freely. The total number of entries into the arms and the sequence of alternations between them were recorded. Memory was assessed based on spontaneous alternation rates, defined as successive entry into three distinct arms, without consecutive repetitions.

#### 4.5.3 New Object Recognition Test

Mice first underwent a training phase in which two identical objects were placed in a corner of the arena, providing the animals with an opportunity to explore freely for 5 min.^22^ After the training session, after a time interval of 1 h (for ST-ORT) or 24 h (for LT-ORT), one of the original objects was replaced with a novel object, and the mice were returned to the arena for a second 5 min exploration period. The time spent exploring each object was recorded. The amount of time spent exploring the novel object compared to the familiar object was used as an indicator of preserved memory. An increased preference for the novel object was interpreted as evidence of intact recognition memory, suggesting that the animal was able to distinguish between the familiar and novel objects.

#### 4.5.4 Object Location Test

The OLT was performed on the same day as the ST-ORT, with a 4 h interval following the training phase. One of the identical objects was relocated to a new position, and the time spent exploring each object was recorded. Greater exploration of the displaced object indicated preserved spatial memory.^22^

#### 4.5.5 Gradual Reduction Inhibitory Avoidance Task

Mice were initially subjected to passive avoidance training, an aversive task designed to assess learning. During training, animals were placed on a platform and, upon stepping down, received a mild electric shock (0.6 mA for 2 s), thereby associating the platform context with the aversive stimulus.^60^ The time taken to step down from the platform was recorded during the training session. Twenty-four h after training, animals were re-exposed to the same environment without the shock to assess contextual memory. The latency to step down was measured during this re-exposure. An increase in the latency to step down indicated that animals had learned to associate the environment with the aversive stimulus, reflecting acquired contextual memory. The cutoff time for stepping down from the platform was 300 s.

### 4.6 Biochemical Analysis

#### 4.6.1 MAO-B Activity Assay

MAO-B activity was assessed in samples from the hippocampus, hypothalamus, cortex, and cerebellum. Measurement of MAO-B activity was performed according to previous methodology,^61^ with adaptations.^62^ Tissues were homogenized manually in a homogenization buffer (0.0168 M Na_2_HPO_4_, 0.0106 M KH_2_PO_4_, 0.32 M sucrose, pH 7.4) in a 1:4 ratio (w/v) to obtain the mitochondrial fraction. Homogenization was followed by centrifugation at 900*g* at 4 °C for 5 min, and the supernatant was subjected to a second centrifugation at 12,500*g* at 4 °C for 15 min. The resulting pellet was resuspended in the homogenization buffer and centrifuged again under the same conditions. The final pellet was solubilized in buffer (0.0168 M Na_2_HPO_4_, 0.0106 M KH_2_PO_4_, 0.0036 M KCl, pH 7.4) and stored appropriately (−80 °C) until further analysis. For the assay, the protein concentration was determined by the Bradford method.^63^

For determination of enzymatic activity, the samples were incubated at 37 °C for 5 min in a buffer solution containing 250 nM clorgyline, a selective MAO-A inhibitor, allowing specific quantification of MAO-B activity. Subsequently, 60 μM kynuramine dihydrobromide was added as substrate, and the reaction was maintained at 37 °C for 30 min. The reaction was stopped by the addition of 10% trichloroacetic acid (TCA), followed by centrifugation at 16,000*g* for 5 min. The supernatant was mixed with 2 mL of 1 M NaOH, and the fluorescence intensity was measured using a spectrofluorometer (excitation: 315 nm; emission: 380 nm). Enzyme activity was expressed as nmol of 4-hydroxyquinoline/mg protein.

#### 4.6.2 AChE Activity Assay

AChE activity was determined according to the method described by Ellman et al. (1961).^64^ Cortex and cerebellum samples were homogenized in Tris-HCl buffer (50 mM, pH 7.4) in a 1:5 ratio (w/v) to obtain the mitochondrial fraction and centrifuged at 900*g* at 4 °C for 10 min. The supernatant was collected for enzymatic analysis. The reaction was conducted in sodium phosphate buffer (0.1 M, pH 7.4) containing 5,5′-dithiobis­(2-nitrobenzoic acid) (DTNB) as a chromogenic agent and acetylthiocholine iodide (AcSCh) as the substrate. The change in absorbance was measured spectrophotometrically at 412 nm over 2 min, reflecting the hydrolysis of AcSCh into the thiocholine, which reacts with DTNB to form a chromogenic complex. AChE activity was expressed as μmol AcSCh/h/mg protein.

#### 4.6.3 Thiobarbituric Acid Reactive Substances (TBARS)

Lipid peroxidation was evaluated by measuring thiobarbituric acid-reactive substances (TBARS) levels, following the method described by Ohkawa et al. (1979),^65^ with slight modifications. Cortex and cerebellum samples were homogenized in Tris-HCl buffer (50 mM, pH 7.4) in a 1:5 ratio (w/v) and the homogenate was centrifuged at 900*g* for 10 min at 4 °C. The resulting supernatant was used for the analysis. An aliquot of the supernatant was incubated with a reaction mixture containing thiobarbituric acid (0.8%), sodium dodecyl sulfate (8.1%), and acetic acid (pH 3.4) at 95 °C for 2 h. After cooling, absorbance was measured at 532 nm using a spectrophotometer. TBARS levels were expressed as nmol of malondialdehyde (MDA) per mg of protein (nmol MDA/mg protein).

#### 4.6.4 Reactive Species (RS) Assay

Quantification of reactive species (RS) was performed by a spectrofluorimetric method using the 2′,7′-dichlorofluorescein (DCFH) assay. This fluorescent marker is widely used due to its ability to cross cell membranes and detect the formation of oxidative products. For analysis, an aliquot of the cortex and cerebellum supernatant prepared in a 1:5 ratio (w/v) was incubated in Tris-HCl buffer (10 mM, pH 7.4), adding DCFH. The resulting fluorescence was recorded 30 min after the addition of the dye, using a spectrofluorometer with excitation at 488 nm and emission at 520 nm. The results were expressed in fluorescence units, reflecting the RS levels in the tissues analyzed.^66^

#### 4.6.5 Nonprotein Thiols (NPSH) Levels

NPSH levels were determined following the method described by Ellman (1959).^67^ The supernatant (S1) of cortex and cerebellum was mixed in a 1:1 ratio (v/v) with 10% TCA to precipitate proteins. The samples were then centrifuged at 900*g* for 10 min, and the pellet was discarded. The resulting supernatant (S2) was used for the quantification of free thiol groups. To initiate the reaction, an aliquot of S2 was added to a solution containing potassium phosphate buffer (1 M, pH 7.4) and 10 mM 5,5′-dithiobis­(2-nitrobenzoic acid) (DTNB). The mixture was incubated at room temperature, and absorbance was measured at 412 nm using a spectrophotometer. NPSH levels were expressed as nmol NPSH per gram of tissue.

#### 4.6.6 NOx Levels

The quantification of nitrates and nitrites (NOx) was performed in cortical and cerebellar samples prepared in a 1:5 (w/v) ratio using a colorimetric reaction based on the reduction of nitrates to nitrites.^68^ Samples were incubated in a reactive solution containing 2% vanadium chloride (dissolved in 5% HCl), 2% sulfanilamide (dissolved in 5% HCl), and 0.1% *N*-(1-naphthyl)­ethylenediamine dihydrochloride. The mixture was maintained at 37 °C for 1 h to allow color development. Absorbance was then measured at 540 nm using a spectrophotometer. NOx concentration was expressed as nmol NOx/mg of protein.

#### 4.6.7 Toxicity Parameters

The analysis of plasma levels of AST and ALT enzymes, as well as urea concentration, was performed using colorimetric enzymatic methods according to the instructions provided in the commercial kits from Labtest Diagnóstica. Blood glucose levels on days 0 and 25 were measured using an Accu-Chek Active glucometer, and body weight was monitored throughout the study.

#### 4.6.8 Protein Determination

For all analyses requiring protein quantification, protein levels were determined using the Bradford method,^63^ a widely established colorimetric assay based on the binding of Coomassie Brilliant Blue G-250 dye to proteins. Briefly, samples were diluted as necessary and mixed with the Bradford reagent, followed by incubation at room temperature. Absorbance was measured at 595 nm using a spectrophotometer, and protein concentrations were calculated based on a standard curve generated with bovine serum albumin (BSA).

### 4.7 RNA Isolation and Quantitative Real-Time PCR (RT-qPCR)

Total mRNA was extracted from the hippocampus using TRIzol followed by mRNA quantification. The cDNA synthesis was performed using a High-Capacity cDNA Reverse Transcription kit according to the manufacturer’s protocol. The amplification was made with the GoTaq qPCR Master Mix using the Stratagene Mx3005P real-time PCR. Gene expressions were normalized using β-actin primer as a reference gene. The genes provided in Table 1 were analyzed.

**Table 1:** Primer Sequences Used

**Gene**	**Sequence 5′–3′**
**β-actin**	F: GTCCCTCACCCTCCCAAAAG
	R: GCTGCCTCAACACCTCAACCC
**BAX**	F: CCACCAGCTCTGAACAGATC
	R: CAGCTTCTTGGTGGACGCAT
**BCL-2**	F: TGGGATGCCTTTGTGGAACT
	R: GAGACAGCCAGGAGAAATCA
**BDNF**	F: TAACGGCGGCAGACAAAAAGACT
	R:GTGTCTATCCTTATGAATCACCAGCCAA
**CAT**	F: GAACGAGGAGGAGAGGAAAC
	R: TGAAATTCTTGACCGCTTTC
**GSK3β**	F: CGGGACCCAAATGTCAAACT
	R: TCCGAGCATGTGGAGGGATA
**IL-6**	F: CCAGAAACCGCTATGAAG
	R: CACCAGCATCAGTCCCAAGA
**NFκB**	F: GCTTTCGCAGGAGCATTAAC
	R: CCGAAGCAGGAGCTATCAAC
**NRF-2**	F: GACGCAGACCCTCTCTTGTC
	R: TAAGCGAACACCAAGCTCCT
**SOD**	F: TGGTGGTCCATGAGAAACAA
	R: GTTTACTGCGCAATCCCAAT
**TNF-α**	F: CATCTTCTCAAAATTCGAGTGACAA
	R: TGGGAGTAGACAAGGTACAACCC

### 4.8 Statistical Analysis

The experimental results were analyzed using GraphPad Prism software version 8.0.1 and expressed as the mean ± standard error of the mean (SEM). One-way ANOVA was employed to detect significant within-experiment effects, followed by the application of Newman–Keuls multiple comparisons. For analysis of toxicity data, a *t*-test was applied. Probability values less than or equal to 0.05 (*p* ≤ 0.05) were considered significant.
